# Moral rhetoric in discrete choice models: a Natural Language Processing approach

**DOI:** 10.1007/s11135-023-01625-8

**Published:** 2023-03-09

**Authors:** Teodóra Szép, Sander van Cranenburgh, Caspar Chorus

**Affiliations:** 1 Engineering Systems and Services, Delft University of Technology, Jaffalaan 5, 2628 BX Delft, The Netherlands; 2 Faculty of Industrial Design Engineering, Delft University of Technology, Delft, The Netherlands

**Keywords:** Moral rhetoric, Discrete choice models, Moral Foundations Theory, Natural Language Processing

## Abstract

This paper proposes a new method to combine choice- and text data to infer moral motivations from people’s actions. To do this, we rely on moral rhetoric, in other words, extracting moral values from verbal expressions with Natural Language Processing techniques. We use moral rhetoric based on a well-established moral, psychological theory called Moral Foundations Theory. We use moral rhetoric as input in Discrete Choice Models to gain insights into moral behaviour based on people’s words and actions. We test our method in a case study of voting and party defection in the European Parliament. Our results indicate that moral rhetoric have significant explanatory power in modelling voting behaviour. We interpret the results in the light of political science literature and propose ways for future investigations.

## Introduction

Choice data is often used to infer people’s underlying preferences about different products, policies or several other subjects. The field of discrete choice modelling focuses on the mathematically rigorous analysis of decision making. Using data on observed choices, the analyst can derive people’s preferences about different attributes in a choice task, such as price or quality. Most decisions in life potentially have a moral dimension, such as consumers considering worker conditions, fair trade, animal welfare or local community when making a purchase, doctors making trade-offs between health outcome and patient experience, or commuters considering how their travel practices affect other commuters or the environment. Morality can be defined as a set of principles that tells whether an action or state of the world is right or wrong.

Therefore, besides the traditional attributes, personality and moral values in particular also often play a significant role in many situations. Moral ’attributes’ are substantially different from non-moral ones. Emotions, intuitions, and decision heuristics play a major role when contemplating trade-offs between them (Haidt 2001; Sunstein 2005; Gigerenzer 2010). These processes are latent not only for the analyst but, in most cases, for the decision-makers too. Recent work regarding a broad range of latent variables, including latent moral motivations, shows that the joint identification of underlying preferences and other latent determinants of decision-making is a very challenging task (e.g. Vij and Walker 2016).

Although progress is being made to advance the identification of such models based on choice data, one obvious potential solution has not received the attention it deserves: the use of additional text data to help identify latent behavioural constructs. One central argument for using text data in choice analysis is that the nuances that are present in free text often cannot be grasped with standard, closed-ended responses (Baburajan et al. 2020)^1^. This is even more relevant when the subjects are abstract and complex phenomena, such as moral values (Boyd et al. 2015). For two main reasons, free text data and language modelling show great promise for understanding how moral values relate to behaviour and choices. First, in the age of the internet and social media, a vast amount of text is generated every day, carrying plenty of information potentially useful for understanding morality and complex decision-making phenomena. Second, language models in the rapidly growing Natural Language Processing (NLP) field approach the human level of text understanding and allow us to quantify qualitative text data in several ways to understand moral values and behaviour better.

This paper proposes a method to combine choice- and text data to infer moral motivations in decision-making contexts. We show how this novel approach can lead to new, subtle insights regarding latent antecedents of moral choice, which would be very difficult “if not impossible”to obtain using traditional choice models based on observed choices only. To test and illustrate our proposed approach, we investigate the voting behaviour of Members of the European Parliament (MEPs) on various moral topics. Voting on such topics, and especially when one votes against their party (defects), is expected to relate to moral motivations. The rest of the paper is organized as follows: Section 2 provides a brief literature review related to our methodological approach. Section 3 describes our general methodology for extracting moral rhetoric of texts, which can be used in various research contexts. Section 4 describes the context of our case study, our data, and the operationalization of the methodology. Section 5 shows the results and discusses their interpretation. Section 6 discusses the limitations of the methodology and future research avenues.

## Related work

Discrete choice models (DCMs) relying on full-fledged Natural Language Processing (NLP) methods to make use of additional text data are not yet used in the literature (van Cranenburgh et al. 2021). A few papers indicate that both NLP methods and additional text data can capture subtleties that were overlooked in the literature before. A recent paper by Pereira (2019), for instance, shows how NLP methods can encode subtle yet important nuances in travel behaviour modelling using DCMs. For example, “student” and “employed” categorical characteristics are rather similar when it comes to departure time choice but dissimilar when it comes to car ownership. In the traditional variable encoding, these are one unit distance from each other. However, word embeddings (i.e. words represented with vectors of real numbers) allow us to encode this subtle difference in choice models. Pereira (2019) does not rely on additional text data but uses the words that are already part of most traditional data (i.e. attributes and personal characteristics). Studies that used free text data^2^ in DCMs include Glerum et al. (2014) who used semi-open questions about different transport modes to include perceptions, and Baburajan et al. (2020) who used open-ended questions to measure attitudes towards shared mobility services.

Studying morality through natural language has been vast and growing in the past decades as increasing computing power allows for higher quality and quantity of text mining and NLP techniques. Most studies in this field rely on the Moral Foundations Theory (MFT, Graham et al. 2009). MFT originates from moral psychology and postulates that people have five innate moral foundations: care/harm, fairness/cheating, loyalty/betrayal, authority/subversion, and sanctity/degradation. According to the theory, these foundations are cross-cultural; they can be found in everyone, only their extent differs across people. Measuring this extent has two main methods. First is a closed-ended questionnaire, the Moral Foundations Questionnaire (MFQ), which asks respondents to what extent different things (e.g. whether or not someone suffers emotionally) affect their moral judgement in a situation. Second is the Moral Foundations Dictionary (MFD), which contains words related to each foundation and direction (i.e. a word can belong to either virtue or vice in the same moral foundation). The first version of MFD was extended several times (Frimer et al. 2019; Hopp et al. 2021; Araque et al. 2020). Operationalization of MFD ranges from word counting methods to sophisticated NLP algorithms. There are two main tools to extract moral foundations from text: MFD (or one of its extended versions, e.g. van den Broek-Altenburg et al. 2020; Kaur and Sasahara 2016; Mutlu and Tütüncüler 2020) and manual (expert) annotation (e.g. Hoover et al. 2020). Furthermore, complex NLP models can be trained using MFD, annotated data, or both, to classify a piece of text into one of the moral foundations (Hoover et al. 2020; Araque et al. 2020). When it comes to interpretation, MFQ is straightforward; scoring high on a moral foundation means a higher emphasis on the given foundation when making a moral judgement. This is not necessarily true for language use. In a political context, interestingly, it was found that although conservatives adhere to loyalty more than liberals, loyalty appears more in liberals’ moral rhetoric (Graham et al. 2009). Although this effect was small, Frimer (2020) found it to be robust. This means that moral rhetoric does not necessarily represent the intrinsic values of a person, and one must be careful with interpreting outcomes.

## Methodology

In this paper, we propose a method of using moral rhetoric as inputs in Discrete Choice Models. Moral rhetoric are the quantified moral dimensions of text data, where a text created by humans has the purpose of projecting an image. This could be honest because one might want to project their actual values. However, it is also possible that one purposefully talks or writes in a specific way to be perceived as endorsing different values. Therefore, moral rhetoric do not necessarily reflect the ’true’ values of the text’s creator, but they do reflect the values the piece of text projects.

**Fig. 1:**
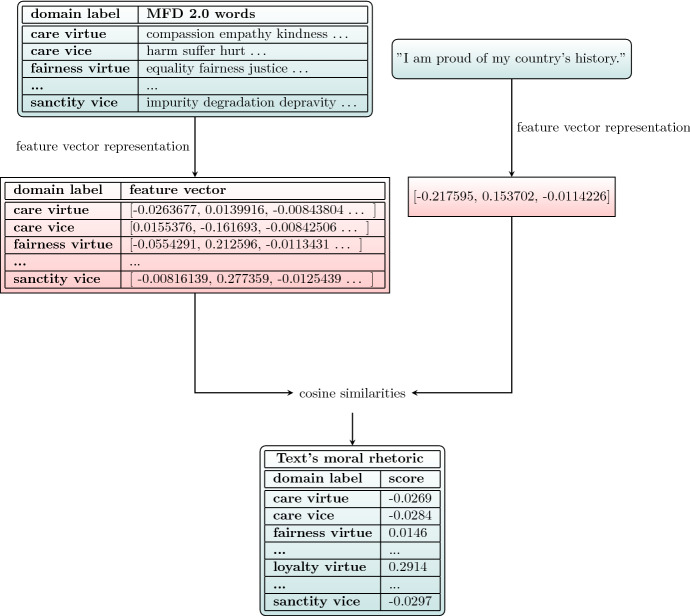
The process of extracting the moral rhetoric for a piece of text. Inputs and output are coloured in blue, and the intermediate steps of the process are coloured in red. The calculation methods are on the corresponding arrows. The example sentence is from the Moral Foundations Questionnaire (MFQ), and we find that the domain of“loyalty virtue”has the highest score. The sentence corresponds to the loyalty foundation according to the creators of MFQ too. (Color figure online)

In order to quantify morality in text data, we need 1, moral text data and 2, an NLP method called feature vector representation. Moral text data is data on different dimensions of morality, such as care, fairness or loyalty. The moral dimensions could be based on Moral Foundations Theory (Graham et al. 2009), Schwartz Values (Schwartz 1992), or Morality-as-Cooperation (Curry et al. 2019), to name a few. Moral Foundations Theory has a large body of literature relating it to text analysis and has a dictionary that was updated several times; thus, without claiming that other definitions of morality are incorrect or less useful, we use the moral domains of MFT in this paper. Feature vector representation means that all words in a text are represented with a vector of real numbers. This can be done in several ways, from more simple such as bag-of-words method^3^ to state-of-the-art Transformers methods (Vaswani et al. 2017). In order to find the moral rhetoric for any piece of text, first, we create feature vectors for all moral domains based on the moral text data. Then we do the same for the piece of text at hand and measure the similarity between the text’s and each moral domain’s vector. To see how similar a text is to each moral domain, we compute the cosine similarity^4^between their feature vectors. This way, a piece of text’s moral rhetoric determines how similar the text is to each of the moral domains. The similarity score can range from -1 to 1. 1 means perfect similarity, 0 means no relation, and -1 means a perfect opposite relation between two vectors. See Fig. 1 for an illustration and the detailed description below.

In order to utilize behavioural data (i.e. choice data), we use the Discrete Choice Model family. According to DCMs, the probability of individual *n* choosing alternative *i* can be generally expressed as:

1 $$\begin{aligned} P_{ni}=Prob(V_{ni}+\varepsilon _{ni} >V_{nj}+\varepsilon _{nj} \ \forall j\ne i) \end{aligned}$$

where $V_{ni}$ is the observed part of the latent continuous variable representing the motivation of decision maker *n* to choose alternative *i*. $\varepsilon _{ni}$ is the random error term, or the unobserved part of the latent motivation.

$V_{ni}$ can be generally characterized as follows.

2 $$\begin{aligned} V_{ni}=f(X_{ni}, S^{m}_{ni}) \end{aligned}$$

where $X_{ni}$ are the attributes of alternative *i* for individual *n*, depending on the choice situation, and $S^{m}_{ni}$ are the scores of the moral domains (i.e. the output of the NLP model). The specification of $f( X_{ni}, S^{m}_{ni})$ depends on the choice task at hand. For instance, one may want to include the moral rhetoric of the decision-makers or the moral rhetoric of different product descriptions, or both at the same time.

## Case study: voting in the European Parliament

To empirically test and illustrate how moral rhetoric can lead to subtle behavioural insights, we use a case study in the field of politics, namely voting in the European Parliament. This section describes our case study, the operationalization of the above methodology, and the results.

One of the most critical decisions is arguably political decision making: elected representatives decide about policies that potentially have a significant effect on many people’s lives. These decisions also often have a moral component: protecting fundamental human rights in foreign countries, helping the poor, investing for the sake of future generations or preserving the environment. In our case study, we examine voting behaviour in the European Parliament (EP) on subjects, such as“Search and rescue in the Mediterranean” or“The impact of Covid-19 on youth and on sport”. In the EP, there are 705 members (MEPs), whom the citizens of the European Union elect in their home countries. Most MEPs belong to one national party in their home country, and these national parties usually join in 7 EP party groups. There are independent representatives too. Although the EP started as a consultative body, it gained power, and by now, it can enact legislation, amend the budget, or censure the Commission. Most voting procedures are not recorded, but roll-call votes are required on final legislation votes and whenever a political group or 30 MEPs request it. In roll-call voting, the vote of each member is recorded. It is established that MEPs most often vote in line with the majority of their EP party group (e.g., Hix 2002; Klüver and Spoon 2015; Lindstädt et al. 2011). The reason for this is twofold: people gravitate towards parties they agree with, and there is party discipline. Therefore, in cases when MEPs defect their party group, we can assume they have a strong reason for it. Thus, besides the votes themselves (three alternatives: for against or abstain), we also rely on party group defection choice data (two alternatives: to defect or not).

### Research background of political voting behavior

MFT has a history of explaining moral value differences across people, and a large amount of literature focuses on political, ideological differences. In the American political system, which is primarily dominated by two main ideologies, liberalism and conservatism, it has been observed that there is a systematic difference between the two groups in terms of moral foundations. According to the initial studies into the subject, it was found that liberals score higher on the so-called *individualizing* foundations, namely care and fairness, while conservatives score lower on these two and higher on the *binding* foundations; loyalty, authority, and sanctity (Haidt and Graham 2007; Graham et al. 2009). This general hypothesis was corroborated by context-dependent studies, such as political text on stem-cell research (Clifford and Jerit 2013) or abortion (Sagi and Dehghani 2014), but also refuted in environmental contexts (Frimer et al. 2015) where liberals used language heavier in sanctity.

In the past few decades, the voting behavior of MEPs has been the subject of several political studies. Hix (2002) hypothesized that MEPs are driven by three main factors: personal preferences, national party discipline and EP party group discipline. The three are often correlated; people with similar beliefs join together in national parties, then national parties with similar agendas join in the EP as party groups. However, there are exceptions in some cases; national parties and EP party groups might disagree, individuals might defect one or both of their parties. These occasions allow for studying which motivations are more important in different situations. Hix (2002) found that the main driving force behind MEPs’ voting behavior is their national party position; measured with distances between MEPs’ EP party group and national party, based on the left-right location and EU-integration location. These were calculated based on a questionnaire where MEPs placed themselves and their parties on this political spectrum. MEP’s individual distance from their EP party group was not significant. The high impact of national party discipline was supported by several studies and extended with additional insights on its reasons (Faas 2002, 2003; Hix 2004; Lindstädt et al. 2011; Klüver and Spoon 2015).

Text data from the documents under votes proved to be valuable assets in several papers in the literature concerned with modelling roll-call vote outcomes, although they did not examine the context of the European Parliament. The goal of these studies is either better prediction (Gerrish and Blei 2010; Kraft et al. 2016; Korn and Newman 2020) or understanding preferences (Lauderdale and Clark 2014; Kim et al. 2018). These latter studies estimate the underlying number of latent dimensions rather than imposing it a priori. This way, they provide insights into how different topics (characterized by sets of words) affect voting behaviour.

### Operationalization of moral rhetoric methodology

Our goal is to test how moral rhetoric in discrete choice models can give nuanced behavioural insights in the context of MEPs voting behaviour. The above literature gives valuable insights based on political science. We test whether similar conclusions can be drawn from a different approach in the field of discrete choice modeling. For moral text data, we use the MFD 2.0 lexicon (Frimer et al. 2019). In a comparison study among the extended versions of MFD, MFD 2.0 was found to be the best in terms of similarity between human-annotated texts and dictionary labels (Mutlu and Tütüncüler 2020). MFD 2.0 is a dictionary of the five moral foundations with corresponding ’virtue’ and ’vice’ words in English, thus resulting in ten moral domains in total. In order to collect choice data (i.e. roll-call voting data), we use the website of the European Parliament. To collect text data from MEPs on their political views, we use their Twitter accounts, which are used as communication channels for political purposes. We collected 328 MEPs’ latest tweets (up to 100) in 2021 April. This data includes short text pieces (up to 140 characters) in 26 different languages. From the European Parliament website, we collected document text data on 24 different voting subjects, such as“Reducing inequalities with a special focus on in-work poverty”or“The EU Strategy for Gender Equality”(see Appendix B for the complete list). Besides the text data, we also collected choice data (i.e. the roll-call votes’), containing whether each MEP voted ’in favour, ’against’, or ’abstain’.

**Fig. 2:**
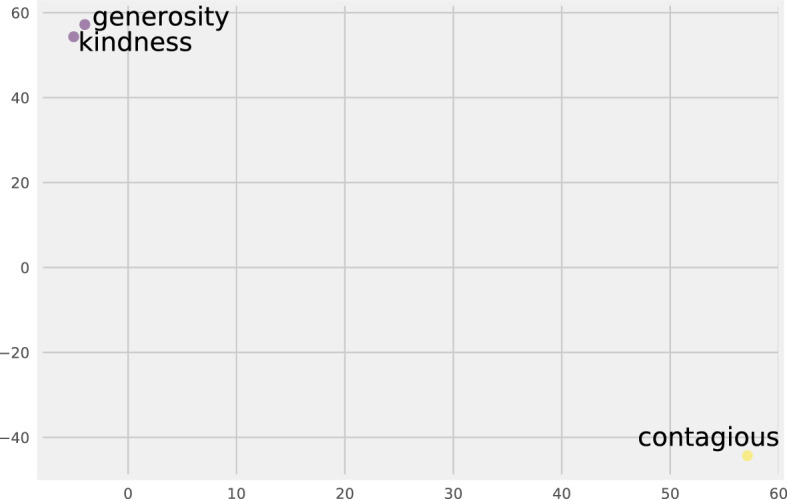
We use 2 dimensions to illustrate the semantic space. The axes do not have interpretation, only the relation among words can be interpreted. For example,“generosity”and“kindness”are closer together in meaning than they are to“contagious”. They also belong to different domains of MFD 2.0 (care virtue and sanctity vice).

We operationalize our proposed methodology (Sect. 3) the following way. We use MFD 2.0 for moral text, thus we create moral rhetoric based on 10 domains. To create feature vector representations, we use a Transformer-based SBERT^5^ (Reimers and Gurevych 2019) model. SBERT is a cutting edge NLP method that allows the words to have a spot in a so-called semantic space. In a semantic space, words (or sentences, or any piece of text) are represented by vectors of real numbers, and words that are closer in meaning are closer in the semantic space as well. See Fig. 2 for a simplified example in two dimensions.

Furthermore, SBERT is able to understand the context of words, meaning that the word“right”has a different vector when the context is human rights and when it is right-wing politics. Its practical advantages are multilingual ability and high speed. We tested this method by extracting moral rhetoric of the sentences of MFQ^6^. We found that in 27 moral rhetoric profiles out of 30, the highest score belonged to the actual foundation the sentence represented (see, for example, a loyalty sentence in Fig. 1). For our case study, we extract moral rhetoric for all tweets, which are then averaged by MEPs to get their individual moral rhetoric. Then MEPs individual moral rhetoric are averaged within parties (both national parties and EP party groups) to get party-specific moral rhetoric. We also extract moral rhetoric for the documents under vote. We use roll-call votes and party defection (casting a different vote than the party majority) as choice data. After a descriptive analysis, we first model EP party defection based on moral rhetoric scores and distances, then we model voting outcome based on document text.

#### Descriptive analysis

It was found in several studies that American conservatives and liberals endorse different moral foundations to a different extent. The American liberal-conservative division in European context is often substituted with left-right division, however in a non-bipolar system partisan differences cannot always be explained by this distinction (Patkós 2023). For instance, Kivikangas et al. (2017) empirically found that in the Finnish political landscape“liberalism-conservatism”cannot interchangeably used with“left-right”in terms of political spectrum division. In a language use examination Proksch and Slapin (2010) also found that EP debate speeches poorly reflect partisan divisions over left-right politics.

**Fig. 3:**
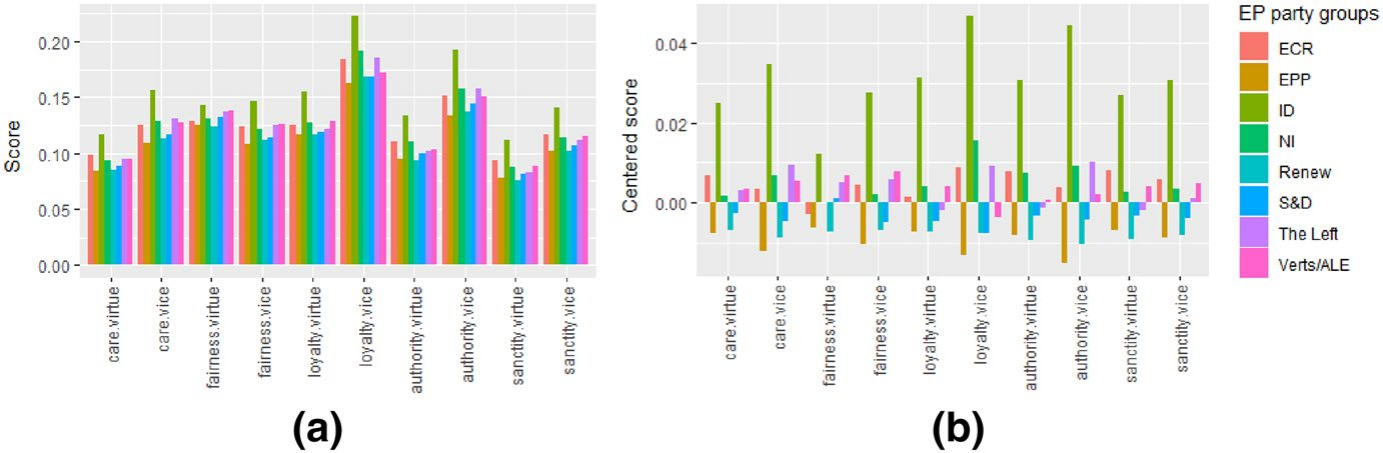
Moral rhetoric of EP parties: Figure** a** shows the average scores of the EP party groups (and independents as“NI”) on the 10 moral domains. Figure** b** shows the centered average scores.

In our case study, we first examine whether moral rhetoric differences can be found in the European political spectrum by plotting the scores of EP party groups based on their members’ tweets. To do this, we extract moral rhetoric for EP party groups by averaging their members’ moral rhetoric. The member’s moral rhetoric are the average moral rhetoric of all their tweets. Figure 3 plots the scores of each party group on the ten moral domains.

From Fig. 3a we can see that the overall pattern is more or less the same for the EP party groups. We find no prominent differences in moral language use nor along the left-right or other political spectrum divisions. The moral rhetoric scores seem to be the same for all parties. This finding is aligned with the line of literature that refutes that the liberalism-conservatism division can be substituted with left-right in the European context (Kivikangas et al. 2017; Patkós 2023). This can be the result of general political discourse: politicians’ topics potentially have a general level of similarity to the MFD 2.0 lexicon. Interestingly, this general similarity is higher for the vice-domain in each foundation, except for fairness. It is also intuitive, as political discourse is often about problems that need solving, and parties might criticize or frame each other negatively (e.g. Turk 2019). The one obvious outlier on Fig. 3a is the ID party group (positioned on the far right). It scores higher on all domains than the other party groups and also shows a somewhat different pattern; their fairness virtue score is relatively low. To see the more subtle differences between party groups, we also plot the centered moral rhetoric (Fig. 3b). Fig. 3b shows that the lowest-scorers on each domain are Renew and EPP, two large parties in the center of the left-right spectrum. This can be interpreted as more radical parties, compared to ones in the center, tend to moralize more to build on people’s (negative) emotions instead of their rational mind (e.g. Salmela and Von Scheve 2017; Turk 2019).

#### EP party defection

First we explore whether MEPs’ and voting documents’ moral rhetoric have explanatory power on defecting one’s EP party group. To do so, we estimate binary logit models where the outcome variable is defecting the EP party group (or not). In our defection analysis we explore two avenues: score-based models, and distance-based models. For both avenues of defection analysis (moral rhetoric score- and distance-based) we first estimate a baseline model, where voting defection is modelled only with EP party groups as explanatory variables. Then we add individual-, party-specific- and document-specific scores and distances in three steps (see Table 1 and 2) for the explanatory variables.

**Table 1:** Score-based models of defection and their included attributes. See corresponding estimated values in Table 3.

	Model 1	Model 2.A	Model 3.A	Model 4.A
ASC and EP party group specific constants	$\checkmark$	$\checkmark$	$\checkmark$	$\checkmark$
Individual moral rhetoric scores		$\checkmark$		$\checkmark$
National party’s moral rhetoric scores			$\checkmark$	$\checkmark$
Document’s moral rhetoric scores				$\checkmark$

**Table 2:** Distance-based models of defection and their included attributes. See corresponding estimated values in Table 4.

	Model 1	Model 2.B	Model 3.B	Model 4.B
ASC and EP party group specific constants	$\checkmark$	$\checkmark$	$\checkmark$	$\checkmark$
Individual distances from EP party group		$\checkmark$		$\checkmark$
National party’s distance from EP party group			$\checkmark$	$\checkmark$
Documents’ moral rhetoric distance from individuals				$\checkmark$

**Fig. 4:**
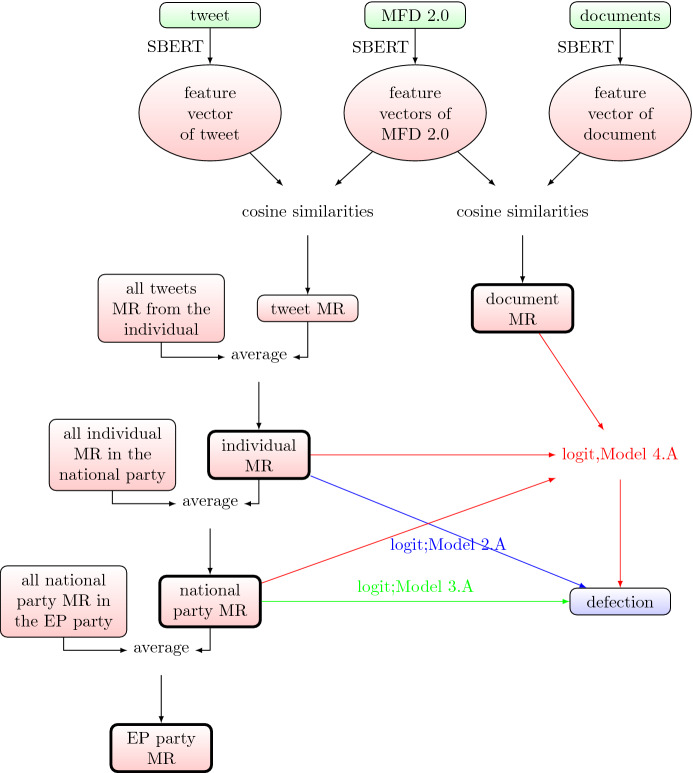
Score-based modelling process corresponding to Table 1 and Eq. A.2. The raw input data are in green, the non-interpretable intermediate steps are in circles, and the calculation methods are displayed on the arrows. Defection is the dependent (or outcome) variable of the modelling process. MR is the abbreviation for“moral rhetoric”, and stands for the 10 domains with corresponding scores illustrated by Fig. 1.

Figure 4 shows the input-output relations corresponding to the score-based modelling process.

**Fig. 5:**
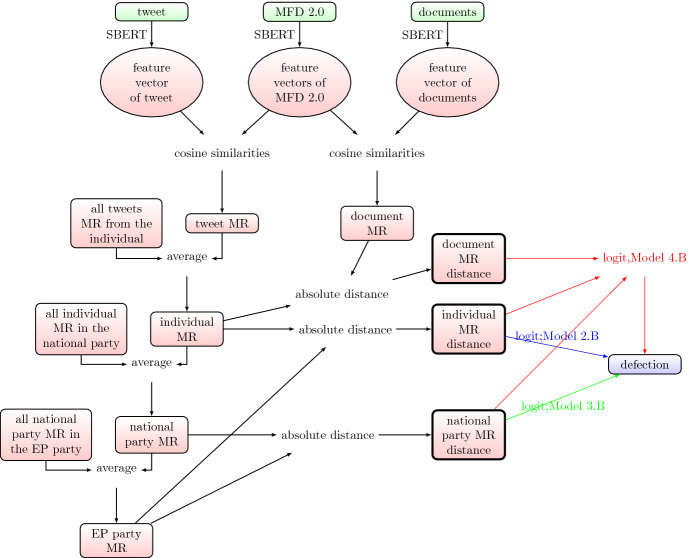
Score-based modelling process corresponding to Table 2 and Eqs. A.3–A.7. The raw input data are in green, the non-interpretable intermediate steps are in circles, and the calculation methods are displayed on the arrows. Defection is the dependent (or outcome) variable of the modelling process. MR is the abbreviation for“moral rhetoric”, and stands for the 10 domains with corresponding scores illustrated by Fig. 1.

Figure 5 shows the input-output relations corresponding to the distance-based modelling process. The key indicators we compare in both modelling approach are model fit and the number of significant parameters.

For more details on the models, see Appendix A.1.

#### Voting outcome

Moving away from party politics, we examine whether legislative texts’ moral rhetoric have explanatory power in voting modeling. In this case, the outcome variable is“for”,“against”, or“abstain”. The explanatory variables are alternative specific constants and moral rhetoric scores of the documents under vote. The modeling has two stages; first, we estimate a model with only alternative specific constants (this model serves as a benchmark), then add the moral rhetoric. We test whether there is a significant improvement in model fit and whether the a priori imposed moral domains are significant for explaining voting results. For the model equations see appendix A.2.

#### Expectations

To formulate our expectations regarding our data analysis, we rely on three main findings from the literature. First, moral foundations can capture political and ideological differences. Second, ideological distances between MEPs’ parties (national and EP) have more explanatory power than MEPs’ individual distance from their EP party when it comes to defecting from their EP party. Third, text analysis of documents has explanatory power in modelling voting outcomes.

***Expectation 1*** We expect that model 3.B has higher explanatory power than model 2.B (in Table 2). Several studies consistently find that political distance between parties has a significant effect on voting defection. We test whether distance based on moral rhetoric has explanatory power when modelling voting defection from the EP party group. Defecting one’s EP party group is naturally assumed to be related to the ideological distance one has from the rest of the party. Interestingly Hix (2002) found that instead of the individual distance, the national party’s distance had more explanatory power when modelling defection. As ideological distance is potentially reflected in one’s moral rhetoric, we expect that ideological distance measured with moral rhetoric based on natural text shows a similar pattern; the national parties’ distance from the EP party group (Model 3.B in Table 4) has more explanatory power than the individual distance (Model 2.B in Table 4).

***Expectation 2*** We expect moral rhetoric to have significant explanatory power in modelling voting outcomes. Modelling voting outcomes based on document text has not been a subject of EP related literature. However, in cases of voting in the US congress and supreme court, text was a good predictor of outcome, and several topic-related preferences were uncovered. For text analysis, these studies used deep learning^7^ methods or topic modelling approach. We test whether the moral rhetoric extracted from the text also have explanatory power. We model voting outcomes with moral dimensions (which correspond to MFT) and expect (some of) them to be significant (model based on Eqs. A.8–A.10).

## Results

In this section we present the results; addressing expectation 1 and 2 in Sects. 5.1, 5.2 accordingly. Section 5.3 summarizes the behavioural findings and limitations of the case study and discusses possible directions for further investigations.

### EP party defection

First, we examine how moral scores of individuals, parties and documents under vote relate to party defection (see the models’ explanatory variables in Table 1). Then, in order to address expectation 1, we examine whether distances between national parties’, individual MEPs’, EP party groups’ and documents moral language use have explanatory power when modelling EP party defection (see the models’ explanatory variables in Table 2).

Table 3 presents the score-based models of defection. For a baseline model, we estimate a binary logit model, where explanatory variables are the alternative specific constant (ASC) for defection and EP party groups (model 1 in Table 3, based on Eq. A.2, assuming $\beta _{m, ind} = \beta _{m,party} = \beta _{m,doc} = 0 \quad \forall m$). The ASC represents the average tendency to vote against one’s party group in the benchmark party group. The benchmark party group is the EPP, the largest one in the EP, positioned in the centre-right. In our data, compared to EPP, ID, Renew, The Left and ECR are more likely to defect, while S &D and The Greens are less likely to defect, assuming everything else is constant. The most likely-to-defect party group is also the one that has the highest scores in their moral rhetoric (i.e. ID party group).

**Table 3:** Score-based models of defection based on moral rhetoric (see Sect. 4.2.1 for details)

	(1)	(2.A)	(3.A)	(4.A)
$ASC_{defect}$	− 2.3099***	− 2.3077***	− 2.30852***	− 2.372***
ID	1.3806***	1.4460***	1.58171***	1.629***
S &D	− 0.9308***	− 0.9895***	− 0.98902***	− 1.043***
Renew	0.5299***	0.4210**	0.29554*	0.255
The Left	0.4765**	0.4420*	0.32553	0.334
ECR	1.0613***	1.1064***	1.19879***	1.200***
The Greens	− 3.8642***	− 3.9524***	− 3.93563***	− 4.021***
care.virtue		− 5.5780		− 4.671
**care.vice**		20.3101*		− 16.285
fairness.virtue		− 1.7170		− 1.261
**fairness.vice**		14.2867*		8.614
loyalty.virtue		5.7068		0.385
loyalty.vice		− 5.5858		− 5.405
authority.virtue		− 5.5437		1.710
**authority.vice**		20.7299**		13.459
sanctity.virtue		− 2.7415		− 3.984
sanctity.vice		− 6.5576		1.412
care.virtue$_{party}$			− 4.12785	− 2.210
care.vice$_{party}$			− 26.89903	− 23.962
fairness.virtue$_{party}$			1.48901	0.409
**fairness.vice**$_{party}$			21.86468*	19.903
**loyalty.virtue**$_{party}$			13.33322*	12.231
loyalty.vice$_{party}$			− 0.08253	− 1.439
*authority.virtue*$_{party}$			− 28.78930*	− 25.967*
**authority.vice**$_{party}$			24.30376*	26.826*
sanctity.virtue$_{party}$			8.49223	7.575
sanctity.vice$_{party}$			− 19.79860	− 22.502
care.virtue$_{doc}$				8.441
**care.vice**$_{doc}$				21.258***
**fairness.virtue**$_{doc}$				13.921***
fairness.vice$_{doc}$				− 9.866
*loyalty.virtue*$_{doc}$				− 10.991***
**loyalty.vice**$_{doc}$				14.536***
*authority.virtue*$_{doc}$				− 21.783***
*authority.vice*$_{doc}$				− 28.148***
**sanctity.virtue**$_{doc}$				14.941*
sanctity.vice$_{doc}$				7.714
Log-Likelihood	− 2038.1	− 2027.5	− 2026.0	− 1974.5
LL(0)	− 4346.0	− 4346.0	− 4346.0	− 4346.0
BIC	4137.4	4203.7	4200.5	4272.5
$Rho^2$	0.53	0.53	0.53	0.55
Number of observations	6270	6270	6270	6270
Number of estimated parameters	7	17	17	37

(1) is the baseline model, in (2.A) individual scores in (3.A) national party scores of moral rhetoric are the explanatory variables. Model (4.A) contains both of the previous moral rhetoric and moral rhetoric of the documents under vote. Moral rhetoric domains that relate to higher than average defection (significant on at least 5% and have positive sign) are highlighted in textbf, those that relate to higher than average party group cohesion (significant on at least 5% and have negative sign) are highlighted in textit. *,**, and ***represents significance on 5%, 1% and 0.1% accordingly

Including individual moral rhetoric scores (model 2.B in Table 3, based on Eq. A.2, assuming $\beta _{m,party} = \beta _{m,doc} = 0 \quad \forall m$) and national party average scores (model 3.A in Table 3, based on Eq. A.2, assuming $\beta _{m, ind} = \beta _{m,doc} = 0 \quad \forall m$) performs similarly in terms of model fit: they show moderate improvement in log-likelihood compared to the baseline model, and the BIC is higher. The significant moral parameters are mostly positive, except for authority.virtue$_{party}$. This means, that for example, someone who scores high on care vice or fairness vice, is more likely to vote against their party group. Those whose national party scores high on authority virtue, are more likely to vote with their party group.

Including both individual and national party scores, along with the documents’ moral rhetoric scores (model 4.A in Table 3, based on Eq. A.2) results in a significantly better model fit in terms of log-likelihood, but in terms of BIC^8^, it does not outperform the benchmark model. Seven out of ten moral domains are significant from the documents’ scores. A positive sign means that subjects that score high on a given domain are more likely to co-occur with a higher than average defection rate. A negative sign correspondingly means that subjects that score high on a given domain are more likely to co-occur with a higher than average level of party group cohesion. Having seven out of ten moral domains significant, we can say that a subject that is heavily loaded with morality (on almost any domain) will be more likely to result in either higher than average defection or, oppositely, cohesion rate. This is intuitive as a morally salient topic can be an incentive to stand up against party groups if one’s own beliefs differ. However, critical moral questions are also likely to be where party groups strongly agree. Model 4.A in Table 3 shows in bold the moral domains more likely to be involved in intra-party-group controversy (i.e. care vice, fairness virtue, loyalty vice, sanctity virtue), and in italic that have the most consensus within parties (i.e. loyalty virtue, authority virtue, authority vice). Table 3 also shows that statistically significant individualizing foundations (i.e. care and fairness) consistently (as individual, national party or document scores) relate to higher than average defection. This is intuitive as those who value or express individualistic foundations verbally are less likely to be driven by group loyalty in moral questions. However, binding foundations (loyalty, authority and sanctity) give a mixed picture: they sometimes relate to cohesion, sometimes to defection. This can be the result of individual MEPs having two parties and their group loyalty being compromised when those do not agree.

**Fig. 6:**
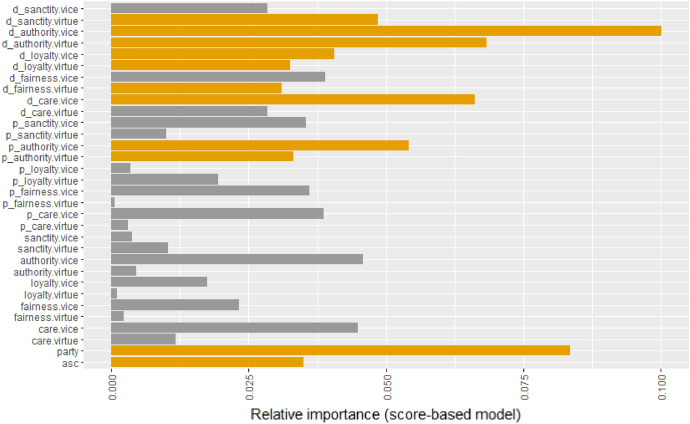
Relative importance of moral rhetoric domains in the score-based full model (model 4.A of Table 3). Significant parameters are signalled with yellow color. (Color figure online)

Figure 6 shows the relative importance of moral rhetoric dimensions when voting against one’s EP party group. We can see that the significant parameters (from document scores and national parties’ authority scores) account for approximately the same fraction of the latent motivation to defect as the alternative specific constant, or the EP party group affiliation.

#### Expectation 1

To address expectation 1, we also model defection based on moral rhetoric distances. Results are presented in Table 4.

**Table 4:** Distance-based models of defection based on moral rhetoric (see Sect. 4.2.1 for details)

	(1)	(2.B)	(3.B)	(4.B)
$ASC_{defect}$	− 2.310***	− 2.269***	− 2.483***	− 2.45661***
ID	1.381***	1.387***	1.565***	1.36797***
S &D	− 0.931***	− 0.942***	− 0.814***	− 0.86512***
Renew	0.530***	0.594***	0.624***	0.71723***
The Left	0.477**	0.564***	0.761***	0.66248***
ECR	1.061***	1.020***	0.914***	0.94051***
The Greens	− 3.864***	− 3.833***	− 3.756***	− 3.79553***
care.virtue		− 3.823		− 5.57490
care.vice		6.367		4.88266
*fairness.virtue*		− 13.466**		− 16.42872***
fairness.vice		6.154		6.24089
loyalty.virtue		− 3.212		2.66383
loyalty.vice		2.915		0.11359
authority.virtue		− 4.026		− 9.02763
authority.vice		3.974		5.97266
sanctity.virtue		14.005		6.55534
sanctity.vice		− 17.419		− 7.91254
$care.virtue_{party}$			− 15.174	− 13.32217
$care.vice_{party}$			4.333	14.14726
*fairness.virtue*$_{party}$			− 27.529***	− 29.14921***
**fairness.vice**$_{party}$			18.769*	18.74683*
*loyalty.virtue*$_{party}$			− 20.372**	− 23.00789**
$loyalty.vice_{party}$			6.252	4.07297
**authority.virtue**$_{party}$			50.430**	53.07479***
$authority.vice_{party}$			4.877	0.03208
**sanctity.virtue**$_{party}$			32.550*	33.88142*
*sanctity.vice*$_{party}$			− 51.132**	− 52.95726**
**care.virtue**$_{doc}$				20.4351**
*care.vice*$_{doc}$				− 54.11249***
$fairness.virtue_{doc}$				1.70708
$fairness.vice_{doc}$				5.00505
**loyalty.virtue**$_{doc}$				11.12370**
*loyalty.vice*$_{doc}$				− 19.09322***
$authority.virtue_{doc}$				− 5.19724
**authority.vice**$_{doc}$				35.6683***
*sanctity.virtue*$_{doc}$				− 27.63144**
**sanctity.vice**$_{doc}$				27.43908*
Log-Likelihood	− 2038.1	− 2026.6	− 2007.6	− 1936.6
LL(0)	− 4346.0	− 4346.0	− 4346.0	− 4346.0
BIC	4137.4	4201.9	4163.8	4196.8
$Rho^2$	0.53	0.53	0.54	0.55
Number of observations	6270	6270	6270	6270
Number of estimated parameters	7	17	17	37

(1) is the baseline model, (2.B) the individual distance-based model, (3.B) the national party distance-based model and (4.B) both of the previous distances and individual MEPs’ distance from document under vote are the explanatory variables. Moral rhetoric domains that relate to higher than average defection (significant on at least 5% and have positive sign) are highlighted in textbf, those that relate to higher than average party group cohesion (significant on at least 5% and have negative sign) are highlighted in textit. *,**, and ***represents significance on 5%, 1% and 0.1% accordingly.

Model 1 in Table 4 is the baseline model without moral distances, exactly the same as model 1 in Table 3.

Model 2.B in Table 4 shows the estimates of our first moral rhetoric distance model: the baseline model extended with the individual moral rhetoric distances between MEPs and their corresponding party groups, following Eq. A.3, assuming $\beta _{m,party} = \beta _{m,doc} = 0 \quad \forall m$. We can see an improvement in the model fit; the log-likelihood ratio test shows a significant difference ($p=0.0109$) from the baseline model. From the moral dimensions, only fairness virtue is significant, with a negative sign. Next, we estimated the defection model based on moral rhetoric distances between the national parties and EP party groups of MEPs (model 3.B of 4) based on Eq. A.3, assuming $\beta _{m,ind} = \beta _{m,doc} = 0 \quad \forall m$. The model shows an even better model fit (*p*-value of log-likelihood ratio test against the baseline model is 0.0000), and six moral domains are significant out of ten. Finally, we estimated the model including the moral rhetoric distances between individual MEPs the documents under vote (model 4.B of 4) following Eq. A.3. This model significantly outperforms the previous ones in terms of model fit, however, the benchmark model (1) has the lowest BIC. From the additional ten document-distance parameters, seven are significant.

**Fig. 7:**
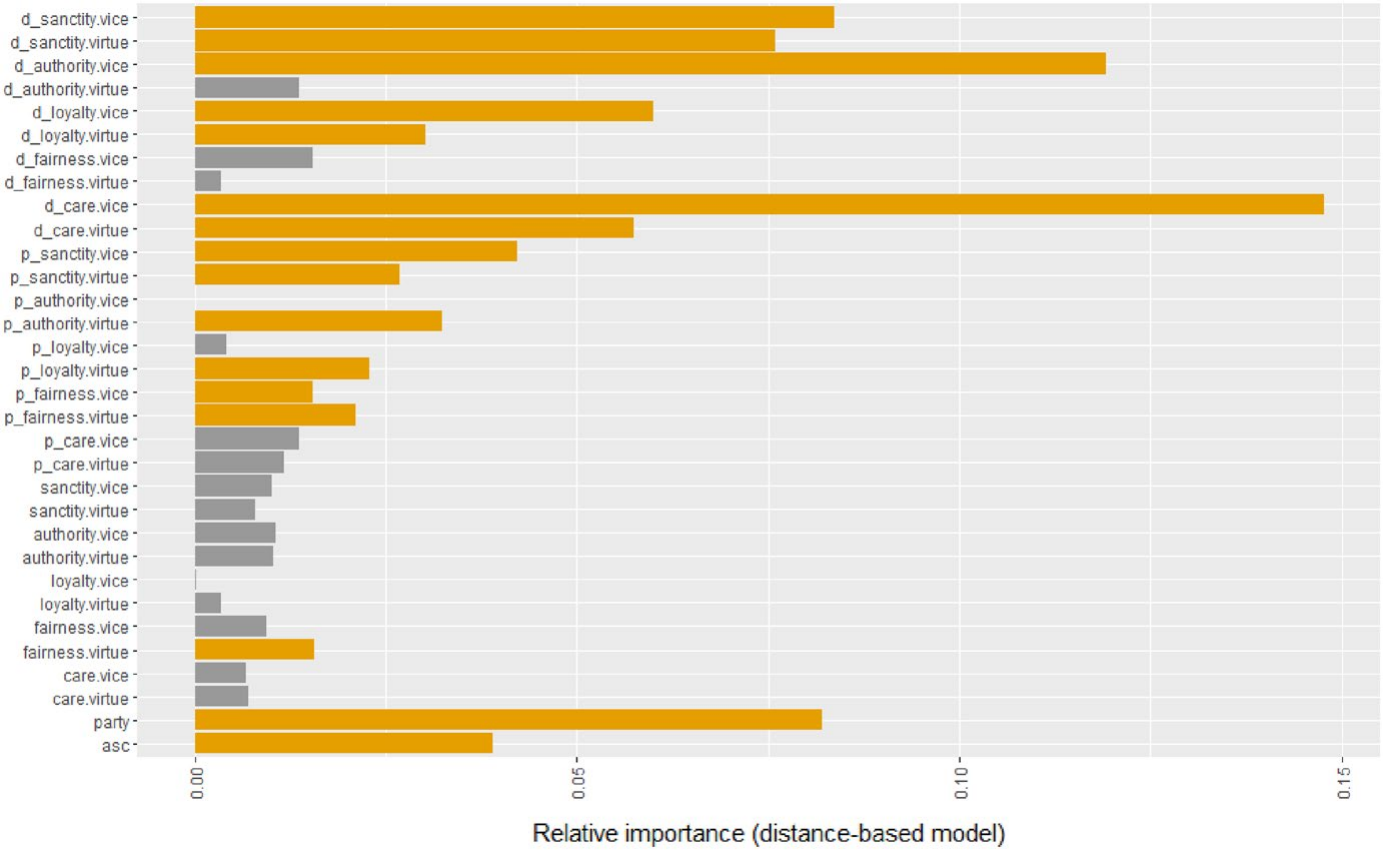
Relative importance of moral rhetoric domains in the distance-based full model (model 4.B of Table 4). Significant parameters are signalled with yellow color. (Color figure online)

Figure 7 shows that the relative importance of moral rhetoric distance dimensions is approximately the same party affiliations’ and often considerably higher than the alternative specific constant.

Based on the distance-based models of EP party group defection (Table 4), we can partly confirm expectation 1. Our results show that more dimensions of the moral-rhetoric-distance are significant, and the model fit is also better when distances are based on party difference (model 3.B of Table 4) instead of individual difference (model 2.B of Table 4). These results could indicate that the subtle ideological differences are captured through language use. Therefore the distance between parties had higher explanatory power than individual distances, similarly to the results found in the literature (e.g. Hix 2002, 2004; Klüver and Spoon 2015). Examining the individual parameters, however, interpretation differs for dimensions with positive and negative weights.

If two moral-rhetoric-profiles have a high distance, that can be attributed to either of two things: the two texts (or people or groups) are covering different subjects (for instance, one talks about decreasing the gender pay gap, and the other about protecting vulnerable animals) or they have different arguments about the same subject (one can frame the same policy as promoting gender equality or as destroying traditional family structures). Thus, high distance is expected to relate to defection. However, our results show that high distance can be related to stronger than average cohesion (negative weights, highlighted in italic in Table 4) as well as to defection (positive weights, highlighted in bold in Table 4).

When a high distance relates to party group cohesion, political forces and other motivations may play a role, thus resulting in a seemingly counterintuitive pattern. We see, for instance, that the fairness virtue dimension has a significant negative (in italic) coefficient in model 2.B of Table 4; thus relates to higher than average EP party group cohesion. This indicates that the more distant someone is from their party group in the fairness virtue dimension, the more likely they vote with their EP party group (with everything else assumed to be constant). One reason this can happen is that despite valuing fairness-related subjects to a different degree, the MEP votes in line with the party group. Then they might want to explain their views toward their constituents. MEPs can have multiple goals that affect voting behaviour, including political ambition. If an MEP intends to climb the legislature’s internal hierarchy, they have a solid incentive to vote with their party groups (Meserve et al. 2009). However, if, for instance, a party group communicates a certain level of fairness-related issues that the MEP finds too low, they might want to reassure their constituents about their values and intentionally tweet more about fairness. This might happen when the MEP scores higher than their party group. The opposite can also happen; if an MEP finds the party group’s communication about a value excessive, they may purposely ignore it on their social media and focus on other issues more relevant for their constituents. In this case, the distance is high because the MEP’s scores are lower than the party group’s. The phenomenon of politicians voting with their party but communicating something different was found by Schwarz et al. (2017) too. In their case study on the Swiss parliament, they find that text analysis reveals more considerable intra-party differences than roll-calls; thus, underlying preferences do not necessarily echo through the choices made by representatives.

In the national party distance model (model 3.B of Table 4), we also find negative coefficients for three moral domains. These can be interpreted slightly differently than in the individual distance model above. The leadership of a party group can exert pressure to ensure national delegations vote inline (Hix et al. 2006). However, national party members might want to appeal to their constituents in their home country and express their different values on their social media, despite voting with the party group due to political pressure. It is also possible that to make their decision acceptable to their followers, they present their voting choice in a frame that resonates more with their followers, which can be very different from the party group’s framing.

In the national party distance model (model 3.B of Table 4), there are positive (in bold) coefficients, too; these mean that the farther the MEP’s national party scores from their EP party group, the more likely that they will defect. As MEPs most often vote with their national parties, it is intuitive that when the moral rhetoric distance is high between a national party and a party group, the delegation will likely defect. It is also possible that when there is tension between a national party and the party group (which can manifest in a high defection rate), their language use will diverge so that they distance themselves from the other. This can happen either by taking opposite stances on specific issues or by discussing different topics online. Tatalovich and Wendell (2018) presents a few examples of how morality policies are typically framed in argumentation, and Clifford and Jerit (2013) empirically shows how stem-cell research is framed relying on the foundation of care or sanctity, depending on whether the argument is“for”or“against”. Slapin and Proksch (2010) looked into the relationship between giving parliamentary speeches and defecting EP party group. They found that those voting against the EP party (often being disciplined by their national party) are more likely to take the floor in parliamentary debates. The reason for this was found to be MEPs demanding speaking time to explain their defection and show their support to their national party and voters on public record. This can be a potential incentive for posting on social media too.

In model 4.B of Table 4 we interacted the document distance (from individual MEPs) with the EP party group’s majority voting “against”. The reason for this is the following. Scoring very different from a document intuitively means that we expect the individual to vote against it or just to vote with their party group. So defection has a different interpretation when the party group preference is “for” and when it is “against”. Our results show that three domains (care vice, loyalty vice and sanctity virtue) follow the intuitive pattern: those who score different from the document are more likely to vote with their party “against”. However, four domains (care virtue, loyalty virtue, authority vice, and sanctity vice) show the opposite pattern. Despite the party group’s preference to vote“against”, when the distance between the document and MEP is high in these four domains, MEPs are more likely to defect; either by voting “for” or “abstain”. This potentially signals that these values play a role in a way which does not echo through words. For instance, if someone scores very low on care virtue, and a document comes to vote which scores very high on care virtue, and the party group discipline is voting “against”, then an individual voting “for” could mean they have values that they do not express on Twitter, likely because of their political agenda.

The domains that are significant when included as EP and national party distance variable, and when included as individual distance from the document too, show a consistent behaviour in the following sense. Loyalty virtue and sanctity vice display a counterintuitive pattern in both cases. Large distance between parties with respect to these domains results in cohesion, and large distance from the document along with party discipline “against” still results in “for” or “abstain”. Thus issues related to these foundations are most likely to stir political pressure from the EP party group’s side, or issues related to these foundations are most likely to stir moral motivations that do not echo through words. On the other hand, sanctity virtue behaves the intuitive way in both cases: large distance between parties relates to defection, and when the distance from the document is high, those whose party group discipline is ‘against’ are significantly less likely to defect. Thus sanctity virtue related issues seem to be where actions and words are most aligned.

### Modelling voting outcome

#### Expectation 2

To address expectation 2, we model voting outcome, meaning whether MEPs voted in favour, against or abstention on a subject. Table 5 shows two multinomial logits with the vote as the dependent variable. Model 1.C only uses ASCs and shows that voting “for” is the most likely choice in our sample and voting “against” is also more likely than abstaining. Next, in model 2.C, we include the moral rhetoric of documents and use alternative-specific weights for them, following Eqs. A.8–A.10. The model fit improves significantly, and the additional 20 parameters are justified based on the BIC. Seven moral domains relate to significantly lower or higher than average “for” rate, and eight domains relate to significantly lower or higher than average “against” rate.

**Table 5:** Model of voting on documents (Sect. 4.2.2 for details), dependent variable has three possible values: “for”, “against” or “abstain”. *,**, and *** represents significance on 5%, 1% and 0.1% accordingly.

	(1.C)	(2.C)
$ASC_{for}$	1.6250***	1.743***
$ASC_{against}$	0.5480***	0.478***
$\beta _{for}$ care.virtue		− 25.684***
$\beta _{for}$ care.vice		− 32.280***
$\beta _{for}$ fairness.virtue		− 8.378**
$\beta _{for}$ fairness.vice		8.259
$\beta _{for}$ loyalty.virtue		13.662***
$\beta _{for}$ loyalty.vice		− 10.608**
$\beta _{for}$ authority.virtue		30.026***
$\beta _{for}$ authority.vice		25.748***
$\beta _{for}$ sanctity.virtue		− 9.285
$\beta _{for}$ sanctity.vice		3.154
$\beta _{against}$ care.virtue		− 27.526***
$\beta _{against}$ care.vice		22.930**
$\beta _{against}$ fairness.virtue		− 22.623***
$\beta _{against}$ fairness.vice		− 13.274*
$\beta _{against}$ loyalty.virtue		0.553
$\beta _{against}$ loyalty.vice		− 9.907*
$\beta _{against}$ authority.virtue		43.219***
$\beta _{against}$ authority.vice		19.534**
$\beta _{against}$ sanctity.virtue		− 24.376***
$\beta _{against}$ sanctity.vice		11.606
Log-Likelihood	− 5498.0	− 4971.4
LL(0)	− 6888.3	− 6888.3
BIC	11013.6	10135.2
$Rho^2$	0.20	0.28
Number of observations	6270	6270
Number of estimated parameters	2	22

Moral rhetoric of documents seem to have explanatory power in the voting model, too, similarly to the score-based defection model (model 4.A in Table 3). The results of Table 5 indicate that the moral rhetoric of documents have explanatory power in modelling voting behaviour as a trinary choice; thus, expectation 2 is met. For example, high fairness virtue score in a proposal is more likely to result in abstention than on average. We see from model 4.A of Table 3 that fairness virtue is also related to a higher than average defection rate. This indicates that fairness virtue is a domain that may stir defection when present in a document under vote, and it materializes in voting abstention. Authority (both virtue and vice), on the other hand, relates to significantly higher “for” and “against” votes, thus resulting in fewer abstentions. This finding is in line with the distinction of individualizing/binding foundations. Fairness is an individualizing foundation; thus, defecting one’s group when a fairness-related issue is at hand is intuitive. Authority is a binding foundation; thus, the individualistic moral motivations to potentially defect play a less significant role.

### Summary and limitations

Overall, our results indicate that moral rhetoric of MEPs and documents under vote have explanatory power in modeling voting behavior. Expectation 1 is partly met and expectation 2 is met. Furthermore, we can gain subtle insights about voting behavior by interpreting the modelling results, such as

- high moral rhetoric scores in individualizing foundations (i.e. care and fairness) of individual MEPs, national parties or documents under vote relate to higher than average party group defection,
- binding foundations (loyalty, authority and sanctity) can relate to both cohesion and defection,
- individualizing foundations in documents more often result in abstentions,
- issues related to loyalty virtue and sanctity vice are most likely to stir political pressure from the EP party group’s side, or issues related to these foundations are most likely to stir moral motivations that do not echo through words,
- sanctity virtue is the moral rhetoric domain where actions and words behave consistently in an intuitive way.

These interpretations must be taken with caution. There are limitations in the case study due to possible selection bias. In our data, those not present at the voting are not included. However, not showing up could also be a strategy similar to abstention, revealing even less information on an MEP’s preferences. Furthermore, we only have data on the tweeting MEPs. MEPs who do not tweet may adopt a different voting strategy than those who do. Lastly, roll-call votes, as they are only part of the legislative decisions, were also argued to cause selection bias Carrubba et al. (2006); however, Hix et al. (2018) found this effect of being negligible in the EP.

#### Future research directions in the voting behavioural context

The above section described several possible reasons for particular signs of our estimated parameters. This paper does not attempt to disentangle the possible effects further. However, there are several ways to go deeper into modelling and answer a wide range of possible research questions regarding moral policymaking. Including, but not limited to:

1. Why do MEPs vote with their national parties as opposed to their EP party group? This can have several practical reasons. For instance, Hix (2004) found that country-specific institutions which reinforce the control national parties can exert over their members increase MEPs’ defection of their EP party groups. Faas (2002, 2003) found that MEPs whose reelection is more dependent on their national parties are more likely to defect. These are national parties that have a centralized candidate selection method, invest more in monitoring their members or are in government in their home countries. Lindstädt et al. (2011) finds that proximity to elections in the home country shifts MEPs’ votes towards their national parties’ when principles of the national party and EP party group conflict. Examining moral rhetoric along these empirically observed effects may shed light on when defection is more likely to be strategic and when is it conviction.
2. How do observations on moral voting behaviour differ across topics? For example, Klüver and Spoon (2015) found that the more salient an issue is to a national party, the stronger the effect of the ideological distance between the national party and its EP party group on MEP defection. In order to gain insight on, for instance, how gender equality related topics differ from the rest, one can model moral rhetoric distances or document scores on divided data sets or include topic-specific categorical variables.
3. How do observations on moral voting behaviour differ across political groups? Different national parties and EP party groups may differ in their relations to moral domains. Individualizing foundations might be more robust predictors in modelling behaviour in progressive parties than in more conservative parties. Such difference, although not in the voting context, was observed in behavioural economics games (Clark et al. 2017).
4. How do behavioural findings change through time? Many studies examined how political rhetoric (e.g. Slapin and Proksch 2008) or voting behavior changes over time (e.g. with election cycles, Lindstädt et al. 2011). Using moral rhetoric in discrete choice models can also shed light on the changing relationship between rhetoric and vote: for instance, is the domain of care virtue (strongly related to health/healthcare) differently related to voting behaviour before and after COVID-19?

Wendell and Tatalovich (2021) argues that some policies are more value-laden than others. There are mixed and pure morality policies. Moral rhetoric extraction is suitable for both mixed and pure morality policies, as, through similarity scores, it is expected to reflect a mixed nature compared to pure morality policies. In the political science direction, other case studies could use moral rhetoric, for instance, examining the voting behaviour of the general public. For this, social media feed or other collected text data, such as a values essay or opinion description about specific topics, could be used.

## Discussion

In this paper, we proposed a method for enriching discrete choice models with moral rhetoric extracted from text, thus connecting the two ways morality can manifest itself: words and actions. We used state-of-the-art Natural Language Processing methods and a well-established moral psychological taxonomy of values, Moral Foundations Theory. We showed in a case study of voting in the European Parliament what subtle insights such moral rhetoric models could provide and discussed other potential applications. Note, however, that in any potential applications, one must be careful with interpretation; there is a complex relationship between moral language, judgement and behaviour, and causal directions are not straightforward. People can have various incentives to hide or obfuscate their true moral judgement when speaking or making decisions, including insecurity, fear of social disapproval, or intention to convince others about something. It is also possible, that in some morally salient situations, people act based on intuition, and create a rational narrative after taking action (Haidt 2001).

The method proposed in this study allows the researcher to study to what extent are words and actions aligned, that can give insights into how strategic behaviour plays a role in various situations. Moral rhetoric can help to identify latent behavioural constructs in more complex discrete choice models such as latent class models or latent variable models. Latent class models are often used to identify classes with moral motivations. Using questionnaires or product attributes was instrumental in finding consumption or behavioural patterns across different classes of people. For instance, Zha et al. (2020) identifies environmentally responsible classes when buying electric appliances, or Langen (2011) identifies groups with different attitudes towards fair trade, organic production and donations when buying coffee. Moral rhetoric may help to identify that in moral situations, groups that display different behavioural patterns can be identified partly based on their language use.

Furthermore, it has been theorized and empirically found that latent variables have a significant effect on what decision-making rule is applied (Hess and Stathopoulos 2013). In moral contexts, this could be even more relevant for two reasons. First, utility maximization is often replaced by moral heuristics (Sunstein 2005; Gigerenzer 2010), which give a wide range of possible decision rules. Second, deep-seated moral values can affect several observable factors, for instance, political affiliation, the way one talks (thus their moral rhetoric) and their choices in moral situations. Thus, the combination of alternative decision-making rules and moral rhetoric can be instrumental in latent variable latent class modelling.

Latent motivations to take one action or another in moral questions potentially have a more complex form than the standard linear additive specification. Different attributes in a choice task might interact with, for instance, how strongly one talks about fairness. Different attributes in a choice task might interact with, for instance, how strongly one talks about fairness. Less obvious interaction effects could also be discovered with, for example, machine learning-assisted methods (see, e.g. Hillel et al. 2019). Such methods can improve the utility specification and thereby improve predictions based on language use while retaining the interpretability of the discrete choice model. Such enriched moral DCMs have the potential to advance persuasion techniques, which have relevance in various applications and research fields, such as marketing, psychology, political science, or policy design.

Future ways for methodological research in moral rhetoric could involve comparison with text classification methods (i.e. the probability of a text belonging to a specific foundation) where the moral rhetoric profiles would be a composite of probabilities and not similarity scores. Future research on the implications of moral rhetoric analysis could be cross-cultural or cross-contextual comparisons: some moral domains may prove to be robust in explaining actions, while some are not. They may vary across cultures, times, or the decision-making situation. Such knowledge can give valuable insights on communication strategies and behavioural phenomena through easily obtainable text data.

## Appendix A: Methodological appendix

### A.1 EP party defection model details

The estimated binary logit models of Table 1 and 2 take the form of

A.1 $$\begin{aligned} P_{defection,i}=\frac{\exp (V_{defection,i})}{1+\exp (V_{defection,i})} \end{aligned}$$

where $V_{defection,i}$ is the latent continuous variable representing a MEP’s motivation to defect, and based on Eq. 2 it is characterized using the following explanatory variables: alternative- and party group-specific constants ($X_{ni}$ from Eq. 2), moral rhetoric and moral rhetoric distances ($S^{m}_{ni}$ from Eq. 2).

In our defection analysis we explore two avenues: score-based models, and distance-based models. First we examine how moral scores of individuals, parties and documents under vote relate to party defection (see the models’ explanatory variables in Table 1).

For the score-based defection analysis, the fully specified model’s latent motivation to defect is characterized as follows:

A.2 $$\begin{aligned} V_{{defection,i}} = & ASC + \sum\limits_{{q \in Q}} {\beta _{q} x_{{q,i}} } \\ & + \sum\limits_{{m \in M}} {\left( {\beta _{{m,ind}} S_{{i,m,ind}} + \beta _{{m,party}} S_{{i,m,party}} + + \beta _{{m,doc}} S_{{m,doc}} } \right)} \\ \end{aligned}$$

where *ASC* is the alternative specific constant for defection, *Q* is a set of party groups, and $x_{q, i}$ is a binary variable, taking the value of 1 when individual *i* is in party group *q*, and 0, when they are not. *M* is the set of moral domains. $S_{i, m, ind}$ is the individual score of individual *i* on moral domain *m*. $S_{i, m, party}$ is the average score of the national party of individual *i* on domain *m*. *S* *m*, *doc* is the score of the document under vote on domain *m*.

For the distance-based defection analysis, the fully specified model’s latent motivation to defect is characterized as follows:

A.3 $$\begin{aligned}{} {} V_{defection,i} & = ASC+\sum _{q \in Q}\beta _{q} x_{q,i} + \sum _{m \in M} \left( \beta _{m,ind} D_{i, m, ind} +\right. \nonumber \\{} & {} \quad \left. + \beta _{m,party} D_{i, m, party} + \beta _{m,doc} \cdot I_{against} \cdot D_{m, doc} \right) \end{aligned}$$

where $I_{against}$ is binary indicator of whether the EP party group of individual *i* prefers “against”^9^. $D_{i, m, ind}$ and $D_{i, m, party}$ are the dimensions of moral rhetoric distances. Moral rhetoric distances are calculated domain-wise; for example, for “care virtue”, the individual distance of MEP *i* from their EP party group is:

A.4 $$\begin{aligned} D_{i, care virtue, ind}=\vert S_{care.virtue, MEP_{i}} - S_{care.virtue, EP party group_{i}} \vert \end{aligned}$$

The distance between the national party and EP party group of MEP *i* is similarly:

A.5 $$\begin{aligned} D_{i, care.virtue, party}= \vert S_{care.virtue, national party_{i}} - S_{care.virtue, EP party group_{i}} \vert \end{aligned}$$

And finally, the distance between the document and MEP *i* is similarly:

A.6 $$\begin{aligned} D_{i, care.virtue, party}= \vert S_{care.virtue, MEP_{i}} - S_{care.virtue, doc} \vert \end{aligned}$$

where $S_{care.virtue}$ is the score corresponding to the“care virtue”domain. Similarly, $S_{m,doc}$ is the score of the document under vote on moral domain *m*.

The latent motivation for non-defection (i.e. voting with the party group) is normalized to be 0.

A.7 $$\begin{aligned} V_{non-defection,i}=0 \end{aligned}$$

### A.2 Voting outcome model details

The model’s systematic components (i.e. latent continuous variables representing MEPs’ motivation to vote for, against or abstention) are specified as follows.

A.8 $$\begin{aligned}{} & {} V_{for}=ASC_{for}+\sum _{m \in M}\beta _{for,m} S_{m, doc} \end{aligned}$$

A.9 $$\begin{aligned}{} & {} V_{against}=ASC_{against}+\sum _{m \in M} \beta _{against, m} S_{m, doc} \end{aligned}$$

A.10 $$\begin{aligned}{} & {} V_{abstain}=0 \end{aligned}$$

where *M* represents the ten moral domains, and $S_{m, doc}$ is the document’s score on moral domain *m*. Abstention is normalized to be zero.

## Appendix B: Subjects of roll-call votes

1. “2019–2020 Reports on Bosnia and Herzegovina”
2. “2019–2020 Reports on Kosovo”
3. “A WTO-compatible EU carbon border adjustment mechanism”
4. “Artificial intelligence: questions of interpretation and application of international law in so far as the EU is affected in the areas of civil and military uses and of state authority outside the scope of criminal justice”
5. “Children rights in occasion of the 30th anniversary of the Convention of the Rights of the Child”
6. “Decent and affordable housing for all”
7. “EU Association Agreement with Ukraine”
8. “Human Rights and Democracy in the World and the EU policy on the matter - annual report 2019”
9. “Human rights and political situation in Cuba”
10. “Human rights situation in Kazakhstan”
11. “Meeting the Global Covid-19 challenge: effects of waiver of the WTO TRIPS agreement on Covid-19 vaccines, treatment, equipment and increasing production and manufacturing capacity in developing countries”
12. “Prisoners of war in the aftermath of the most recent conflict between Armenia and Azerbaijan”
13. “Promoting gender equality in science, technology, engineering and mathematics (STEM) education and careers”
14. “Reducing inequalities with a special focus on in-work poverty”
15. “Regulatory fitness, subsidiarity and proportionality - report on Better Law-Making 2017, 2018 and 2019”
16. “Search and rescue in the Mediterranean”
17. “Strengthening the single market: the future of free movement of services”
18. “Systematic repression in Belarus and its consequences for European security following abductions from an EU civilian plane intercepted by Belarusian authorities”
19. “The EU Strategy for Gender Equality”
20. “The adequate protection of personal data by the United Kingdom”
21. “The gender perspective in the COVID-19 crisis and post-crisis period”
22. “The impact of Covid-19 on youth and on sport”
23. “The right to disconnect”
24. “EU accession to the Istanbul Convention and other measures to combat gender-based violence”

## Footnotes

1 In their recent work, Baburajan et al. (2022) find that although Topic Modeling is suitable to extract information from open-ended responses, discrete choice models estimated using closed-ended questions perform better than those using the open-ended questions.

2 By free text, we refer to a piece of text that is not the result of a closed-ended question. Free text can be either the response to an open-ended question or something that a person expresses on their own initiative, for example, social media posts.

3 The bag-of-words representation carries information on the words and their number of occurrence in a piece of text. It disregards the grammar and word order.

4 Cosine similarity is a measure of similarity between two sequences of numbers viewed as vectors. For two vectors,*A*, *B* and the angle between them $\theta$, the cosine similarity is calculated with the following formula: $\cos (\theta )=\frac{\sum _{i=1}^{n}A_i B_i}{\sqrt{\sum _{i=1}^{n}A_i^2} \sqrt{\sum _{i=1}^{n}B_i^2}}$, where $A_i$is the *i*th element of vector *A*.

5 We use the model ’paraphrase-multilingual-mpnet-base-v2’ Reimers and Gurevych (2020).

6 We used MFQ30, which can be found at https://moralfoundations.org/questionnaires/.

7 The goal of such deep learning models is accurate classification, and no interpretation or reasoning is provided in these black-box approaches.

8 Note that BIC measures model fit while penalizing a high number of parameters. The models of Table 3 and 4 are not targeted to find the best model for this particular case but to illustrate how moral rhetoric scores and their distances can be used in choice models. Thus, we did not merge scores and distances to find better BIC.
